# Supplementary material for: Raman Spectral Signatures of Serum-Derived Extracellular Vesicle-Enriched Isolates May Support the Diagnosis of CNS Tumors
Source: Cancers (Basel). 2021 Mar 19;13(6):1407. doi: 10.3390/cancers13061407 (PMC8003579; doi:10.3390/cancers13061407)
Supplement: Supplementary file 1 [file cancers-13-01407-s001.zip › Cancers_supplementary_materials_proofreading.docx]

Raman Spectral Signatures of Serum-Derived Extracellular Vesicle-Enriched Isolates May Support the Diagnosis of CNS Tumors

Matyas Bukva, Gabriella Dobra, Juan Gomez-Perez, Krisztian Koos, Maria Harmati, Edina Gyukity-Sebestyen, Tamas Biro, Adrienn Jenei, Sandor Kormondi, Peter Horvath, Zoltan Konya, Almos Klekner and Krisztina Buzas

**Supplementary materials**

**
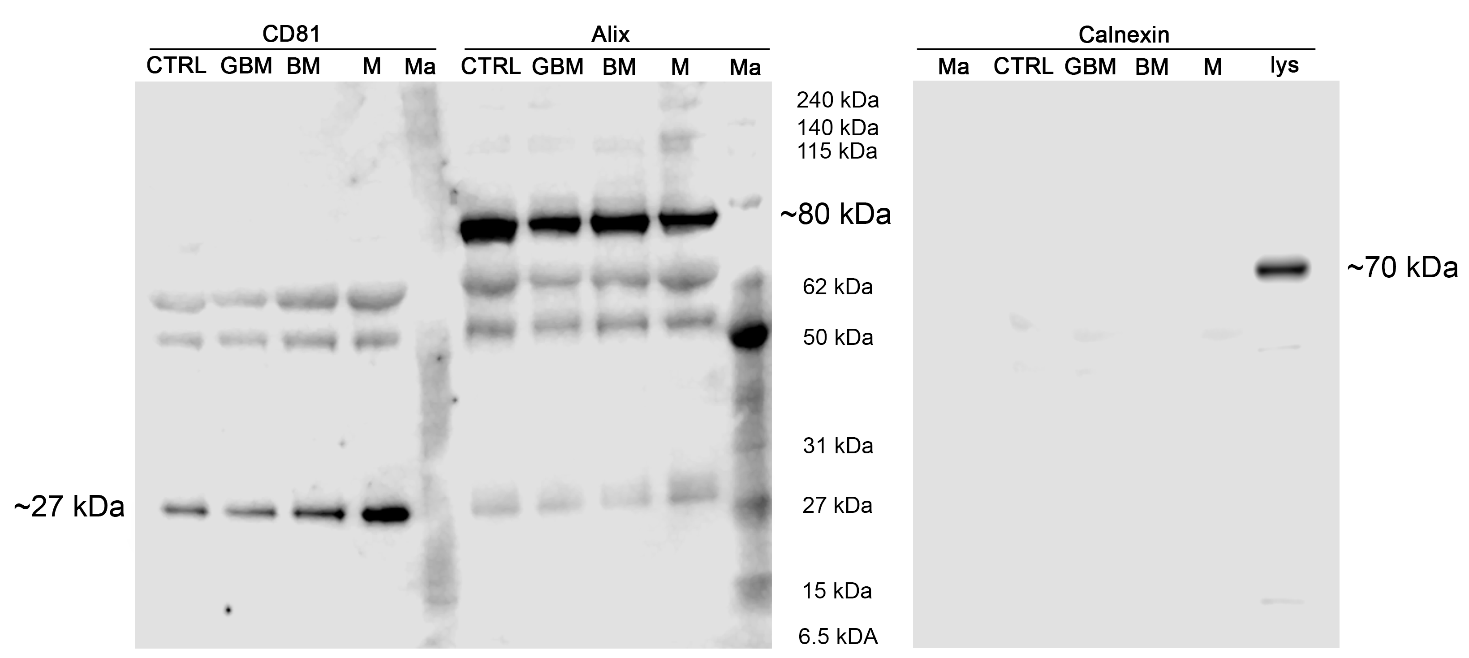
Figure S1**. Original images of the Western blot analysis. This figure contains original Western blot images of the three sEV protein markers (Alix, CD81, calnexin). (Abbreviations: Ma, protein ladder).


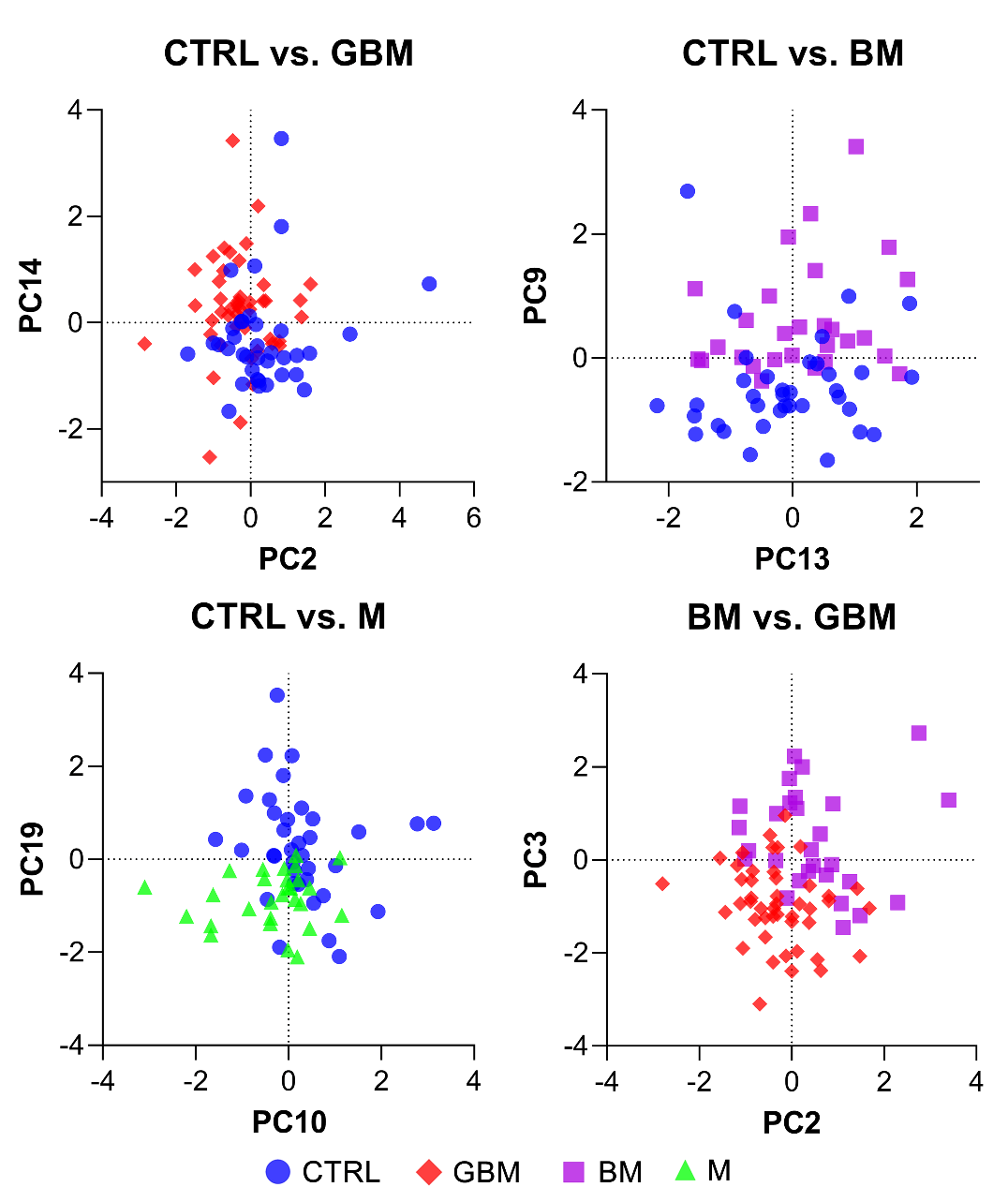
**Figure S2.** PCA score plots of the selected PCs. This figure represents the score plots of the selected PCs in every comparison.

**Table S1**. Tabular form of the discriminative spectral differences. The table contains the discriminative spectral differences presented in Figure 7.

**Table S2**. The baseline-corrected Raman spectroscopic data. The table contains the baseline-corrected spectral data of the sEV samples from the four patient groups.
